# Multi-centre randomised controlled trial of a smart phone-based event recorder alongside standard care versus standard care for patients presenting to the Emergency Department with palpitations and pre-syncope - the IPED (Investigation of Palpitations in the ED) study: study protocol for a randomised controlled trial

**DOI:** 10.1186/s13063-018-3098-1

**Published:** 2018-12-29

**Authors:** Matthew J. Reed, Neil R. Grubb, Christopher C. Lang, Rachel O’Brien, Kirsty Simpson, Mia Padarenga, Alison Grant, Sharon Tuck

**Affiliations:** 1 Emergency Medicine Research Group Edinburgh (EMERGE), Department of Emergency Medicine, Royal Infirmary of Edinburgh, 51 Little France Crescent, Edinburgh, EH16 4SA UK; 2 College of Medicine and Veterinary Medicine, University of Edinburgh, The Chancellor’s Building, 49 Little France Crescent, Edinburgh, EH16 4SB UK; 3 Department of Cardiology, Royal Infirmary of Edinburgh, 51 Little France Crescent, Edinburgh, EH16 4SA UK; 4 Edinburgh Clinical Research Facility, Epidemiology and Statistics Core, University of Edinburgh, Western General Hospital, Crewe Road South, Edinburgh, EH4 2XU UK

**Keywords:** Ambulatory electrocardiography monitoring, Cardiac arrhythmias, Palpitations, Pre-syncope

## Abstract

**Background:**

Palpitations and pre-syncope are together responsible for 300,000 annual Emergency Department (ED) attendances in the United Kingdom (UK). Diagnosis of the underlying rhythm is difficult as many patients are fully recovered on ED arrival; and examination and presenting electrocardiogram (ECG) are commonly normal. The only way to establish the underlying heart rhythm is to capture an ECG during symptoms. Recent technology advances have led to several novel ECG monitoring devices appearing on the market. This trial aims to compare the symptomatic rhythm detection rate at 90 days of one such smart phone-based event recorder (AliveCor Heart Monitor and AliveECG) with standard care for participants presenting to the ED with palpitations and pre-syncope and no obvious cause in the ED.

**Methods/Design:**

This is a multi-centre hospital ED / Acute Medical Unit (AMU) open label, randomised controlled trial. Participants will be recruited in 10 tertiary and district general hospitals in the UK. Participants aged ≥ 16 years presenting with an episode of palpitations or pre-syncope with no obvious cause and whose underlying ECG rhythm during these episodes remains undiagnosed after clinical assessment will be included. Participants will be randomised to either: (1) the intervention arm, standard care plus the use of a smart phone-based event recorder; or (2) the control arm, standard care. Primary endpoint will be symptomatic rhythm detection rate at 90 days. A number of secondary clinical, process and cost-effectiveness endpoints will be collected and analysed. Analysis will be on an intention-to-treat basis.

**Discussion:**

The Investigation of Palpitations in the ED (IPED) study aims to recruit 242 participants across 10 hospital sites. It will be the first study to investigate the ability of a smart phone-based event recorder to detect symptomatic cardiac rhythms compared to standard care for ED patients with palpitations and pre-syncope with no obvious cause in the ED. This smart phone event recorder will allow ED patients who have presented with palpitations or pre-syncope to record their ECG tracing if they have a further episode and may increase the rate of underlying rhythm diagnosis.

**Trial registration:**

ClinicalTrials.gov, NCT02783898. Registered on 26 May 2016.

## Background

Palpitations (the noticeable pounding, fluttering or irregular beating of the heart) and pre-syncope (the sudden onset of a sense of impending loss of consciousness) are together responsible for 1.0% of Emergency Department (ED) visits (300,000 annual ED attendances in the UK) [1, 2]. They are less likely to be due to serious arrhythmia than syncope (sudden onset of brief loss of consciousness) and are more likely due to conditions such as atrial fibrillation (AF) and supraventricular tachycardia (SVT). Patients suffering syncope lose consciousness during the episode, meaning patient-activated ECG recorders are not suitable for this population.

Diagnosis of the underlying rhythm is difficult as many patients are fully recovered on ED arrival and examination and presenting electrocardiogram (ECG) are commonly normal. Once captured, the symptomatic rhythm underlying many episodes (about 9/10) is found to be due to benign causes such normal sinus rhythm, sinus tachycardia or frequent ectopics (extra or skipped heart beats) [3]. However, around 1/10 patients do have a cardiac arrhythmia as their symptomatic rhythm [3].

The only way to establish the underlying heart rhythm is to capture an ECG during symptoms. Many patients go for years without diagnosis due to the difficulty in capturing the underlying heart rhythm. Recommended first line investigation of 12-lead ECG [4] and conventional ambulatory monitoring (Holter or event monitoring) [5, 6] are of limited efficacy due to the infrequency of symptoms in many patients [5, 7–10]. Most patients are discharged from the ED and asked to represent or call an ambulance should they get further symptoms in the hope of increasing the chances of capturing the episode on a standard 12-lead ECG. In one US study, the admission rate for this patient group was 24.6% [2].

If patients are referred to cardiology services for assessment, investigation usually starts with a Holter monitor but non-compliance and lack of extended monitoring reduces diagnostic yield to < 20% [11]. Traditional event recorders, external continuous loop recorders and implantable loop recorders are expensive and not recommended for a patient group who rarely have malignant arrhythmias and may have prolonged periods between episodes.

Recent technology advances have led to several novel ECG monitoring devices appearing on the market. The pocket sized AliveCor Heart Monitor and AliveECG phone/tablet app is a monitoring device that requires the patient to trigger the ECG recording. It is available for both Apple and Android mobile and tablet operating systems and was CE marked in January 2015 [12]. With minimal training, two fingers from each hand are placed on the monitor (which can be connected to the back of a smart phone) for 30 s to take an ECG recording, which is transmitted wirelessly to the app, analysed and synchronised to an encrypted server. The patient can then alert their healthcare professional to allow their ECG to be viewed securely [12].

The device is supported for clinical use by a National Institute for Health and Care Excellence (NICE) technology appraisal [12] and was initially developed for detecting AF, for which the automatic diagnostic algorithm has excellent sensitivity (96.6%) and specificity (94%) for correctly interpreting AF versus normal sinus rhythm [13]. The Arrhythmia Alliance [12] distributed AliveCor Heart Monitors to 1500 people of all ages. Only one returned their monitor because it caused them to worry and check their heart rate too often. Of recordings, 26% showed a previously undetected arrhythmia and 5% were advised to see their doctor urgently. They reported that the AliveCor Monitor was ‘easy for people of all ages to use, and could save money for the NHS when compared with the cost of standard NHS ECG recordings.’ Older people were also noted to be regular users of mobile technology and gave positive feedback about the system [12]. This is supported by previous work from our group [14].

There have been several studies investigating the use of smart phone-based event recorders including AliveCor for population screening for AF in various settings [15–19] and feasibility for other rhythm disorders [3, 20]. While AliveCor has now undergone assessment against conventional care for AF detection [21], it has yet to be assessed against standard care for the broader investigation of palpitations and arrhythmia assessment. There have been no studies in an acute or ED population, where large numbers of patients present [2].

We believe this smart phone event recorder will allow ED patients who have presented with palpitations or pre-syncope to record their ECG tracings if they have further episodes, increasing the rate of underlying rhythm diagnosis. If the patient only records benign symptomatic rhythms then they can be reassured, given advice about managing the episodes when they occur and can be discharged from further investigation. Patients with cardiac arrhythmia can referred to specialist care.

### Study aims

#### Primary aim

1. To compare the symptomatic rhythm detection rate at 90 days of a smart phone-based event recorder (AliveCor Monitor) compared to standard care for participants presenting to the ED with palpitations and pre-syncope with no obvious cause in the ED.

A ‘symptomatic rhythm’ will be any ECG rhythm recorded during an episode of palpitations or pre-syncope. This can be either via the AliveCor Heart Monitor ECG or through standard care. Any ECG recorded through the AliveCor Heart Monitor ECG and sent to the study team by the participant will be defined as symptomatic.

#### Secondary aims

1. To investigate the symptomatic rhythm detection rate for cardiac arrhythmia detection at 90 days of a smart phone-based event recorder compared to standard care for participants presenting to the ED with palpitations and pre-syncope.
2. To compare the time to detection of symptomatic rhythm versus standard care.
3. To compare the time to detection of cardiac arrhythmia (rhythm that is not sinus rhythm/sinus tachycardia/ectopic beats) versus standard care.
4. To compare the number of participants treated or (planned for treatment) for cardiac arrhythmia versus standard care.
5. To investigate participant satisfaction and monitor compliance.
6. To compare the cost of symptomatic rhythm detection at 90 days of a smart phone-based event recorder compared to standard care for participants presenting to the ED with palpitations and pre-syncope.
7. To compare serious outcomes at 90 days (all cause death and major adverse cardiac events [MACE] = myocardial infarction, life-threatening arrhythmia, insertion of a pacemaker or internal cardiac defibrillator, insertion of pacing wire) in participants using a smart phone-based event recorder compared to standard care.

## Methods

### Design

This is a multi-centre hospital ED / AMU open label, randomised controlled trial of participants aged ≥ 16 years presenting with an episode of palpitations or pre-syncope and whose underlying ECG rhythm during these episodes remains undiagnosed after ED assessment. Participants will be followed up at 90 days using hospital records, contacting participant GP and also by contacting the participants themselves. This study is expected to take around 18 months to complete from first participant recruited to last participants’ 90-day follow-up.

### Setting

EDs and AMUs of 10 tertiary and district general hospitals in the UK.

### Primary endpoint

1. Symptomatic rhythm detection rate of a smart phone-based event recorder for symptomatic rhythm detection at 90 days versus standard care.

### Secondary endpoints

1. Symptomatic rhythm detection rate of a smart phone-based event recorder for cardiac arrhythmia detection at 90 days versus standard care.
2. Time to detection of symptomatic rhythm using a smart phone-based event recorder versus standard care.
3. Time to detection of symptomatic cardiac arrhythmia (rhythm that is not sinus rhythm/sinus tachycardia/ectopic beats) using a smart phone-based event recorder versus standard care.
4. Number of participants treated or (planned for treatment) for cardiac arrhythmia in participants using a smart phone-based event recorder versus standard care.
5. Participant satisfaction and monitor compliance.
6. Cost-effectiveness analysis.
7. Serious outcomes at 90 days: all cause death and MACE (myocardial infarction, life-threatening arrhythmia, insertion of a pacemaker or internal cardiac defibrillator, insertion of pacing wire).

### Population

A total of 242 consecutive participants aged ≥ 16 years presenting with an episode of palpitations or pre-syncope and whose underlying ECG rhythm during these episodes remains undiagnosed after ED assessment shall be recruited into the study. Recruitment will last around 15 months.

### Inclusion criteria

1. Participant aged ≥ 16 years;
2. Participant presenting with an episode of palpitations or pre-syncope with no obvious cause;
3. Participant’s underlying ECG rhythm during these episodes remains undiagnosed after clinical assessment.

### Exclusion criteria

1. Prior diagnostic ECG;
2. Palpitations or pre-syncope present during an admission ECG;
3. Frequent episodes (i.e. at least once a day);
4. Participants aged < 16 years;
5. Previous participation in the study;
6. Alcohol / illicit drugs / seizure / stroke / transient ischemic attack / head trauma / hypoglycaemia as presumptive cause;
7. Inability or unwilling to give informed consent;
8. Participants with recent (i.e. within three months) myocardial infarction, severe heart failure (NYHA class 4) or unstable angina;
9. Participants unwilling or unable to use the AliveCor Heart Monitor and AliveECG app;
10. Participants without a compatible smart phone or tablet;
11. Participants with cardiac pacemakers or other implanted electronic devices;
12. No telephone number for follow-up;
13. Participant in custody.

### Participant selection and enrolment

The research team, where it is locally agreed that they are part of the clinical care team, will screen and identify potential participants using triage information and clinical or electronic records in the ED or the AMU. In this case, it is anticipated they would identify potential participants and make the first approach. Any member of the clinical team who has received general and trial specific training and is on the delegation log may also identify participants in this way. If the research team are not considered to be part of the direct care team locally, activities carried out before consent (including identification) will be carried out by a member of the direct care team. Where the researcher is not considered to be part of the care team, the researcher should ask a member of the direct care team to identify suitable participants and ask permission from the participant to be approached by the researcher to discuss participation.

If the potentially eligible participant fulfils the study eligibility criteria, a member of the study research team (or direct care clinician if suitably trained) will take written consent. The participant is assessed by the direct care team to establish if he/she is competent and has capacity to consent. This assessment will be documented in the medical notes, i.e. this participant is eligible and capable of providing written informed consent. Participants lacking capacity who are unable to provide consent will not be approached to take part in the study. The participant (and, if present and appropriate, their accompanying relative) will be given a Participant Information Sheet, which will explain the aims of the study and the potential risks and benefits of the study procedures/tests. The participant will be given enough time to consider the study and ask questions regarding their participation in the study. For some participants this could be as much as 1 h but for others may only be 10–15 min. If the participant agrees, informed consent will be confirmed with a signature on the study consent form. The original consent form will be filed in the Investigator Site File (ISF), the participant will receive a copy of this document and a copy will be filed in the participant’s medical notes. Potential, eligible participants who are able to express their consent and able to complete the consent form will be asked to provide written consent. The recruiting direct care clinician or member of the research team will do this. A witness will sign to confirm that all the study information was given and the participant consented to taking part in the study for participants who are able to express their consent but unable to sign.

### Screening for eligibility

There is a requirement to ensure Good Clinical Practice for published studies and to include a Consort diagram [22] of numbers of participants with the study condition during the study period who for one reason or another were not enrolled in the study. In order to determine the number of eligible but not recruited participants during the study period, the ED patient record of all potentially eligible participants will be interrogated by a member of the study research team (if part of the participant’s direct care team). An anonymised log will be kept for patients who were screened for the study and those who were subsequently found to be ineligible or who were not recruited. Non-recruited but potentially eligible participants will be identified by a daily search of all acute patient admissions records to assess for potential selection/recruitment bias. Ineligible patients will not be recorded.

### Randomisation

Participants will be equally distributed between the two study arms with 121 participants in each arm. In order that participants are randomly allocated to a treatment group, while maintaining a balance across the treatment groups, randomisation will be by permuted block randomisation. Randomisation codes will be generated; blinded envelopes will be prepared and labelled in accordance with the randomisation list for each participating site. Permuted random block sizes of 30 will be used. Randomisation codes will be held by each recruiting research team in an accessible area. Participants eligible for inclusion should be randomised by taking the next lowest consecutively numbered envelope.

### Treatment allocation

Participants will be allocated either to: (1) the intervention arm, consisting of standard care plus the use of a smart phone-based event recorder; or (2) the control arm, consisting of standard care, depending on the group allocated in the study envelope. If a participant wishes to withdraw from the study, they will be removed. We will establish whether they consent to allow the use of all data collected up to the date of removal.

### Study interventions

**Fig. 1:**
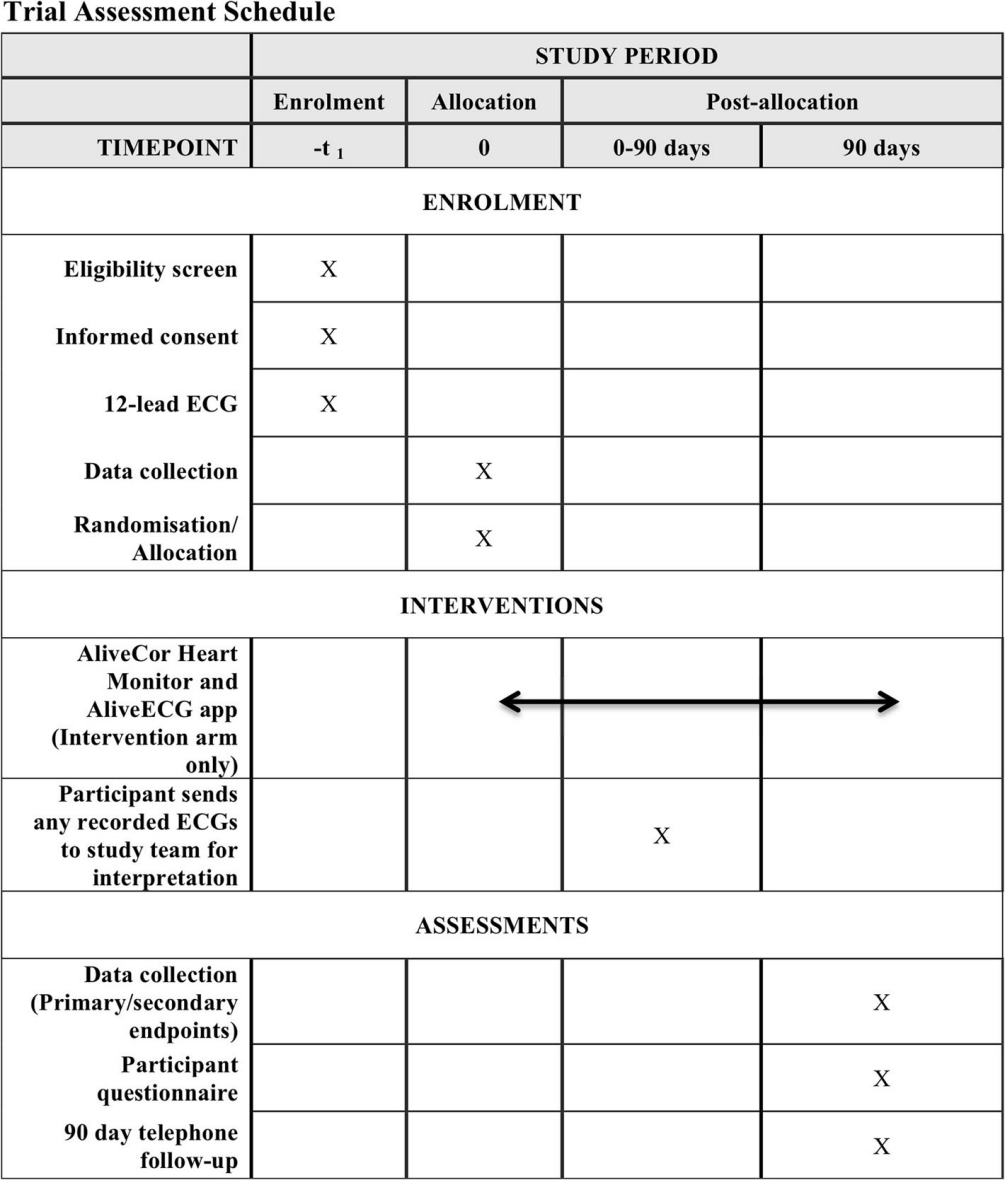
Trial assessment schedule

Figure 1 details the Trial Assessment Schedule. Potentially eligible ED and AMU participants will be identified and assessed for study inclusion by the attending clinician. Written consent will be taken and participants will have a case report form (CRF) completed and a 12-lead ECG taken if not already performed by the clinical team. All intervention arm participants will be given an AliveCor Heart Monitor and trained in the use of the device and app in the ED or AMU by the research team. Control arm participants will receive no other research intervention. Participants in both groups will be admitted, referred or discharged by the treating clinician according to current local hospital protocols. Ongoing treatment and investigation in both groups will be according to current local hospital protocols. Participants in both groups will be followed up at 90 days through hospital record systems (paper or electronic depending on local policy), GP records and by telephone by the local study team. Participants will also be asked to complete a standardised written questionnaire and will receive a follow-up telephone call from the local study team enquiring about symptoms and contact with medical services. Participants will also be asked about satisfaction and compliance with the heart monitor.

Participants allocated to the intervention arm may be contacted by telephone by the local research team shortly after randomisation in the event of them requiring further training or setting up of the device. For example, this may be necessary if a participant does not have their app store password with them in the ED or AMU. If a local electronic patient record system is available, then a research alert will be placed on this stating that the participant has consented to be part of the IPED study to avoid inadvertent unauthorised co-enrolment with other research studies.

If a participant allocated to the intervention arm gets an episode of palpitations or pre-syncope and is able to record an AliveCor Heart Monitor ECG during the episode, the participant will email the ECG recorded by the AliveCor app directly to the coordinating Edinburgh research team at a convenient time to the participant and to a secure nhs.net email address. This email includes a PDF attachment of the ECG tracing along with the participant’s AliveCor app login (which will be their IPED study number - no identifiable participant data will leave the local site, only the participants email address and IPED study number will appear), time and date of recording, and ECG recording. AliveCor collects non-sensitive usage data through a third-party service called Mixpanel. Participants will not be asked to send their ECGs to AliveCor directly for analysis. The AliveCor app rhythm analysis algorithm will automatically report any ECG recorded by the AliveCor app as Normal, Atrial Fibrillation or Unclassified.

The duty Consultant Emergency Physician at the coordinating Edinburgh centre along with a trial team Emergency Physician will review the ECG and will contact the local study team to arrange follow-up if required. In cases of disagreement, the central cardiology team were contacted for further opinion. If specialist follow-up of the ECG tracing is not required then the local study team will write to the participant informing them of this (Patient ECG follow-up letter normal v2.0 24,082,016 and Patient ECG follow-up letter abnormal v2.0 24,082,016) and asking them to arrange follow-up with their general practitioner who will be contacted with the report (GP Follow up Letter v2.0 24,082,016). The participant and GP will not be contacted further should participants record other similar ECGs that similarly do not require specialist follow-up.

The local study team will alert the participant immediately by telephone and refer them urgently to their local ED or cardiac electrophysiology service (as per local protocol) if the participant records a serious cardiac arrhythmia during the study period, i.e.:

- ventricular fibrillation (VF);
- ventricular tachycardia (VT) (it will be assumed that this is symptomatic given the participant has chosen to record an ECG during the episode);
- complete or third-degree heart block;
- second-degree heart block type II (it will be assumed that this is symptomatic given the participant has chosen to record an ECG during the episode);
- pause > 6 s;
- symptomatic bradycardia < 40 beats per minute.

### Participant follow-up

Participants will be asked to log any symptoms along with the time and date, type of symptom and whether they were able to record an ECG during the symptoms, in a participant symptom diary. They will return this diary to the local research team along with the participant satisfaction and compliance questionnaire, and smart phone-based event recorder at the end of the 90 days in a pre-paid, stamped, addressed envelope. Participants will be phoned at 90 days by the local study team to remind them to complete the participant satisfaction and compliance questionnaire and to return this to the local study team with the symptom diary and smart phone-based event recorder. The compliance questionnaire is designed to capture whether participants had the device with them and were able to record a heart tracing during symptoms. This will be entered onto the study database by the local study team.

### Data collection

Participants will have a CRF completed during index hospitalisation, comprising demographic, historical and examination characteristics. An ECG will also be taken and stored. Participant contact details will also be confirmed including a telephone number and email address. Once a participant has been randomised, the baseline CRF will be sent to the local study team office. The information on the CRF will be entered into a specially designed password protected online accessed secure database (REDCAP; http://www.project-redcap.org), the server of which is held within the University of Edinburgh. No participant identifiable information will leave the recruiting NHS hospital or be entered onto REDCAP. Participants will be identified on REDCAP by study number alone.

### Statistics and sample size calculation

Using a symptomatic rhythm detection rate at 90 days of 25% [12] versus standard care (10%), we estimate 110 participants in each group would have 80% power to determine a 15% improvement in symptomatic rhythm detection. We will recruit an extra 10% in each arm to allow for drop-out (i.e. 121 participants in each arm). Our ED sees around 500 eligible participants a year. Assuming a 50% recruitment rate, we estimate we will need to recruit for 15 months.

### Proposed analysis

Descriptive analysis of participant characteristics shall be presented split by study arm. Baseline to 90 day change in diagnostic yield between the two study arms will be analysed using two sample t-tests or non-parametric equivalent as appropriate. Log-rank tests and Kaplan–Meier curves shall be used to examine if the smartphone recorder has an effect on detecting symptomatic rhythm and cardiac arrhythmia separately up to 90 days versus standard care. Categorical variables will be compared using a χ^2^ test (and χ^2^ test for trend if appropriate). All participants will be analysed on an intention to treat basis.

### Study conduct; protocol amendments

Any changes in research activity, except those necessary to remove an apparent, immediate hazard to the participant in the case of an urgent safety measure, must be reviewed and approved by the Chief Investigator. Amendments to the protocol must be submitted in writing to the appropriate REC and local R&D for approval before participants being enrolled into an amended protocol. A Data Monitoring Committee has not been established.

### Protocol violations and deviations

Prospective protocol deviations, i.e. protocol waivers, will not be approved by the sponsor and therefore will not be implemented, except where necessary to eliminate an immediate hazard to study participants. If this necessitates a subsequent protocol amendment, this should be submitted to the REC and local R&D for review and approval if appropriate. Protocol deviations will be recorded in a protocol deviation log and logs will be submitted to the sponsors every three months. Each protocol violation will be reported to the sponsor within 24 h of becoming aware of the violation.

### Reporting, publication and notification of results

Ownership of the data arising from this study resides with the study team. On completion of the study, the study data will be analysed and tabulated, and a clinical study report will be prepared in accordance with ICH guidelines.

The results of our research will be disseminated in the following ways:

1. Summary disseminated to NHS Lothian communication systems including EMERGE intra- and Internet sites and all participating sites;
2. A media summary;
3. Presentation at local and national educational, clinical and research meetings and international research meetings;
4. Publication in peer-reviewed journals;
5. Research report disseminated to NHS Lothian R&D, NHS Research Scotland, and CHSS.

The clinical study report will be used for publication and presentation at scientific meetings. Investigators have the right to publish orally or in writing the results of the study.

## Discussion

The IPED study will recruit 242 participants across 10 hospital sites. It will be the first study to investigate the ability of a smart phone-based event recorder to detect symptomatic cardiac rhythms compared to standard care for ED patients with palpitations and pre-syncope with no obvious cause in the ED. This smart phone event recorder will allow ED patients who have presented with palpitations or pre-syncope to record their ECG tracing if they have a further episode and may increase the rate of underlying rhythm diagnosis.

## Trial status

The trial opened to recruitment in July 2016 in Edinburgh and by early 2017 there were 10 UK sites participating. Recruitment is anticipated to run until December 2017 with trial completion by mid 2018. As of November 2017, 88% of the study population was recruited. This is protocol version number 3.0, dated 09 October 2016.

## Abbreviations

AF: Atrial fibrillation
AMU: Acute Medical Unit (or equivalent)
CHSS: Chest, Heart & Stroke, Scotland
CRF: Case report form
ECG: Electrocardiogram
ED: Emergency Department
EMERGE: Emergency Medicine Research Group of Edinburgh
EPR: Electronic patient record
ICH: International Conference on Harmonisation
MACE: Major adverse cardiac event
NYHA: New York Heart Association
PDF: Portable document format
REC: Research Ethics Committee
SVT: Supraventricular tachycardia
VF: Ventricular fibrillation
VT: Ventricular tachycardia
